# O.4.5-7 Inactive by force? Barriers and motivation to exercise in older adults during a pandemic

**DOI:** 10.1093/eurpub/ckad133.215

**Published:** 2023-09-11

**Authors:** Zsofia Eva Szekeres, Noelia Agustín-Sierra, Lisa Zaidell, Katya Mileva, Rita de Oliveira

**Affiliations:** Cardiff Metropolitan University, UK; London South Bank University; Complutense University of Madrid; zszekeres@cardiffmet.ac.uk; London South Bank University; London South Bank University; London South Bank University

## Abstract

**Purpose:**

The lockdown restrictions over the Covid-19 pandemic have accelerated the ongoing ‘physical inactivity pandemic’ and significantly affected physical and mental well-being in older adults (Eime et al., 2022; Pérez et al., 2021; Sjöberg et al., 2022, Peterson et al., 2021). This study aimed to elucidate the factors that limit or facilitate physical activity in inactive older adults. The main objectives were to identify: (1) the perceived barriers that influence motivation to exercise or physical activity; (2) physical activity patterns when living under lockdown restrictions; (3) how the restriction impacted the motivation and exercise behaviour in older adults.

**Methods:**

Participants were 24 older adults (M = 74 years, SD = 5.0) either physically active or inactive before the pandemic. Semi-structured interviews and self-reported physical activity measures were taken during lockdown and followed up at eight weeks. Transcripts were analysed using thematic analysis.

**Results:**

The three main themes were: sense of purpose for being physically active, routes for engagement, and inactive by force. The sense of purpose was found to be a key source of motivation to exercise influenced by both the belief in the importance of exercise and the affective valence participants assigned to it. Active participants valued exercise due to its health benefits and enjoyment. With the closing of exercise settings, they missed the social context that gave their exercising a purpose. However, both active and inactive participants found purpose in walking outdoors for exercise, as a response to lockdown.

**Conclusions:**

Overall, we suggest that enjoyment and the maintenance of physical function should be targeted in all recommendations and interventions because these are the benefits of exercise most highly valued by older adults. Exercise recommendations and interventions at each socio-ecological level should be tailored specifically to older adults. Supporting older adults to find a valued activity that suits their needs and preferences should be the focus of these strategies. Inactive older adults should be intensively supported by their social and physical context to implement more physical activity in their lives. Our main recommendations include emphasising the importance and benefits of physical activity, supporting and enabling exercise for all ages and levels of mobility.

